# Oocyte meiosis-coupled poly(A) polymerase α phosphorylation and activation trigger maternal mRNA translation in mice

**DOI:** 10.1093/nar/gkab431

**Published:** 2021-05-28

**Authors:** Jun-Chao Jiang, Hua Zhang, Lan-Rui Cao, Xing-Xing Dai, Long-Wen Zhao, Hong-Bin Liu, Heng-Yu Fan

**Affiliations:** MOE Key Laboratory for Biosystems Homeostasis and Protection and Innovation Center for Cell Signaling Network, Life Sciences Institute, Zhejiang University, Hangzhou 310058, China; College of Animal Sciences, Zhejiang University, Hangzhou 310058, China; MOE Key Laboratory for Biosystems Homeostasis and Protection and Innovation Center for Cell Signaling Network, Life Sciences Institute, Zhejiang University, Hangzhou 310058, China; MOE Key Laboratory for Biosystems Homeostasis and Protection and Innovation Center for Cell Signaling Network, Life Sciences Institute, Zhejiang University, Hangzhou 310058, China; MOE Key Laboratory for Biosystems Homeostasis and Protection and Innovation Center for Cell Signaling Network, Life Sciences Institute, Zhejiang University, Hangzhou 310058, China; Center for Reproductive Medicine, Cheeloo College of Medicine, Shandong University, Jinan 250012, China; MOE Key Laboratory for Biosystems Homeostasis and Protection and Innovation Center for Cell Signaling Network, Life Sciences Institute, Zhejiang University, Hangzhou 310058, China; Key Laboratory of Reproductive Dysfunction Management of Zhejiang Province, Assisted Reproduction Unit, Department of Obstetrics and Gynecology, Sir Run Run Shaw Hospital, School of Medicine, Zhejiang University, Hangzhou 310016, China

## Abstract

Mammalian oocyte maturation is driven by strictly regulated polyadenylation and translational activation of maternal mRNA stored in the cytoplasm. However, the poly(A) polymerase (PAP) that directly mediates cytoplasmic polyadenylation in mammalian oocytes has not been determined. In this study, we identified PAPα as the elusive enzyme that catalyzes cytoplasmic mRNA polyadenylation implicated in mouse oocyte maturation. PAPα was mainly localized in the germinal vesicle (GV) of fully grown oocytes but was distributed to the ooplasm after GV breakdown. Inhibition of PAPα activity impaired cytoplasmic polyadenylation and translation of maternal transcripts, thus blocking meiotic cell cycle progression. Once an oocyte resumes meiosis, activated CDK1 and ERK1/2 cooperatively mediate the phosphorylation of three serine residues of PAPα, 537, 545 and 558, thereby leading to increased activity. This mechanism is responsible for translational activation of transcripts lacking cytoplasmic polyadenylation elements in their 3′-untranslated region (3′-UTR). In turn, activated PAPα stimulated polyadenylation and translation of the mRNA encoding its own (*Papola*) through a positive feedback circuit. ERK1/2 promoted *Papola* mRNA translation in a 3′-UTR polyadenylation signal-dependent manner. Through these mechanisms, PAPα activity and levels were significantly amplified, improving the levels of global mRNA polyadenylation and translation, thus, benefiting meiotic cell cycle progression.

## INTRODUCTION

During mouse oocyte meiosis, immature oocytes develop into mature oocytes, coupled with follicle development and are eventually arrested at this stage. Active transcription events occur during this process with the volume of oocytes increasing gradually (1). Precursor mRNAs are processed into mature mRNAs through various events in the nucleus, including capping at the 5′-end, alternative splicing, cleavage and poly(A) addition at the 3′-end (2). Cleavage and polyadenylation of the 3′-end are mediated by multiple proteins and complexes, including cleavage and polyadenylation specificity factor (CPSF), poly(A) binding proteins and poly(A) polymerases (PAPs) (3–5). Nuclear PAPs generally add a short poly(A) tail to primary mRNAs, which are then transported to the cytoplasm where they maintain a low level of translation. Once an oocyte resumes meiosis, cytoplasmic mRNA polyadenylation, translational activation and degradation occur, along with germinal vesicle (GV) breakdown (GVBD) and two successive M-phases (MI and MII) (6). The short poly(A) tail of these mRNAs is further extended by cytoplasmic PAPs, resulting in a longer poly(A) tail, which contributes to higher levels of mRNA translation (7,8).

Mouse oocytes are an ideal model for studying post-transcriptional cytoplasmic mRNA polyadenylation regulation, as fully grown GV-stage oocytes are transcriptionally silent, and meiosis is driven by mRNA translation products stored in the cytoplasm. mRNA translation is generally repressed in GV stage-arrested oocytes and activated during oocyte maturation (9,10). This transition is mediated by the meiosis-coupled mitogen-activated protein kinase (MAPK) cascade and cytoplasmic polyadenylation element (CPE)-binding protein-1 (CPEB1) (11–13). Cytoplasmic polyadenylation relies on two key elements in the 3′-UTR of mRNA: the polyadenylation signal (PAS), which binds to the CPSF complex, recruits PAP, and is indispensable for mRNA polyadenylation, and the CPE, which recruits CPEB1 (14,15). CPEs repress translation at the GV stage but promote translation after GVBD owing to CPEB1 phosphorylation by CDK1, MAPK3 and MAPK1 (also known as ERK1/2) (12,16,17). ERK1/2 triggers the global elevation of translational activities in oocytes during meiotic maturation (11). However, although many transcripts do not contain CPEs in their 3′-UTRs, they undergo translational activation. Biochemical studies suggest that ERK1/2 is capable of directly regulating PAP activities via phosphorylation (18); however, the physiological significance of this mechanism has not been investigated.

In somatic cells, the CPSF complex plays a vital role in nuclear mRNA polyadenylation by recruiting PAP and binding to mRNA (19). CPSF4, an important subunit of the CPSF complex, is responsible for PAS binding (20). However, a recent study has revealed that CPSF4 is located in both the nucleus and cytoplasm of fully grown oocytes, where it participates in mRNA cytoplasmic polyadenylation (21). Upon overexpression of the dominant negative CPSF4 mutant, which lacks a PAS-binding domain, meiosis exhibited severe defects (21). These results suggest that the CPSF complex is indispensable for mRNA cytoplasmic polyadenylation and normal oocyte maturation. Nevertheless, PAPs that mediate cytoplasmic mRNA polyadenylation together with CPSF during mouse oocyte maturation have not yet been identified.

Several cytoplasmic PAPs have been examined, including PAPD4 (also known as GLD2), PAPD5 and PAPD7 (22). Although GLD2 is the cytoplasmic PAP present during oocyte maturation in *Xenopus*, *Gld2*-knockout mice remain fertile, and *Gld2* deletion has a minor effect on cell cycle-related mRNA polyadenylation during mouse oocyte maturation (23,24). PAPD5 and PAPD7, two human orthologs of yeast Trf4, participate in the nuclear surveillance of multiple nuclear target RNAs (25). However, a recent study identified PAPD5 and PAPD7 as the enzymes responsible for mRNA guanylation. These enzymes produce a mixed poly(A) tail with an intermittent non-adenosine residue that shields mRNA from rapid deadenylation (26). Hence, GLD2, PAPD5 and PAPD7 are dispensable in mRNA polyadenylation during mouse oocyte maturation.

There are at least three forms of nuclear PAPs in mammalian cells: PAPα, PAPβ and PAPγ. They are also known as canonical PAPs and are considered the only PAPs that control co-transcriptional polyadenylation in the nucleus (22). However, their potential function in cytoplasmic polyadenylation has not yet been investigated. PAPβ is only expressed in the testes and is essential for spermatogenesis (27), whereas PAPγ is specifically active during tumorigenesis, exhibiting monoadenylation activity toward small RNAs (28). In contrast, although PAPα is ubiquitously expressed, its *in vivo* function has not yet been reported.

In this study, we provide evidence that nuclear PAPα is released into the ooplasm following GVBD, mediating the cytoplasmic polyadenylation of mRNA during mouse oocyte maturation. Furthermore, ERK1/2 increases maternal mRNA translation by phosphorylating PAPα at three previously unidentified sites. In addition, ERK1/2 and PAPα promote the translation of mRNAs encoding PAPα, thereby forming a positive feedback loop during meiosis. These novel regulatory pathways enhance global mRNA polyadenylation and translation and are important for the normal progression of meiosis.

## MATERIALS AND METHODS

### Mice

Wild-type (WT) ICR mice were purchased from the Zhejiang Academy of Medical Science, China. Mice were housed under specific-pathogen-free conditions in a constant environment, including 20–22°C, a 12/12-h light/dark cycle, 50–70% humidity, and food and water provided *ad libitum*. Animal care and experimental procedures were conducted according to the Animal Research Committee guidelines of Zhejiang University.

### Oocyte culture

Three-week-old female mice were primed with 5 IU of pregnant mare serum gonadotropin; fully grown oocytes were collected in M2 medium (M7167; Sigma-Aldrich) 44 h later. These oocytes were then cultured with or without MAPK/ERK kinase 1 and 2 (MEK1/2) inhibitor U0126 (20 μM) in M16 medium (M7292; Sigma-Aldrich), which was covered with mineral oil (M5310; Sigma-Aldrich) and incubated at 37°C in a 5% CO_2_ atmosphere.

### Plasmid construction

cDNAs encoding human PAPα and CPSF4 were subcloned into FLAG-tagged and HA-tagged expression plasmids, respectively. Non-inhibitable CDK1 was constructed by substituting T14 and Y15 of human CDK1 with an alanine and a phenylalanine residue, respectively. Continuously activated MEK1 was obtained by mutating S218 and S222 of human MEK1 to aspartic acid residues.

### *In vitro* transcription and preparation of mRNAs for microinjections

Expression vectors (pRK5 and pDEST) were linearized with HindIII and transcribed *in vitro* using an SP6 mMESSAGE mMACHINE kit (Invitrogen, AM1450). Transcribed RNAs were subjected to phenol/chloroform extraction and ethanol precipitation. Except for 3′-UTR_m_*_Papola_*, 3′-UTR_m_*_Pabpn1l_* and 3′-UTR_m_*_Btg4_*, all mRNAs were *in vitro*-polyadenylated using a poly(A) tailing kit (Invitrogen, AM1350).

### Microinjection of mRNAs and small interfering RNAs (siRNAs)

All microinjections were performed using an Eppendorf TransferMan NK2 micromanipulator. To inhibit spontaneous GVBD, fully grown GV oocytes were harvested in M2 medium containing 2 μM milrinone. Approximately 5–10 pL of 500 μg/ml mRNAs or 20 μM siRNAs were microinjected into the ooplasm of oocytes. For overexpression experiments, 5–10 pL *in vitro* transcription products from the empty vector (pDEST-FLAG, 500 μg/ml) was microinjected into the control oocytes. Injected oocytes were cultured in M16 medium containing 2 μM milrinone at 37°C and 5% CO_2_ for 12 h to allow the translation of microinjected mRNAs or removal of siRNA-targeted transcripts. The oocytes were then further cultured in milrinone-free M16. GVBD and PB1 emission rates were examined at the indicated time points after milrinone release. Immunofluorescent detection of MI and MII spindle was performed at 8 and 14 h after release from milrinone, respectively. The siRNA sequences used are listed in Supplementary Table S1.

### Poly(A) tail (PAT) assay

First, 150 oocytes were used for total RNA extraction using an RNeasy Mini kit (Qiagen). The obtained RNAs were then incubated with poly(dT)12–18, oligo(dT)-anchor and T4 DNA ligase prior to reverse transcription (RT) to generate PAT cDNA. The polyadenylation state of mRNAs was analyzed using PCR with sequence-specific primers (sequences listed in Supplementary Table S1), oligo(dT)-anchor primer and PAT cDNA as templates. A0 was PCR amplified using gene-specific primers (Supplementary Table S1). PCR conditions were as follows: 30 s at 94°C, 20 s at 58°C and 40 s at 72°C for 35 cycles. Polyadenylation states of the PCR products were analyzed on a 2% agarose gel, and images were captured during exposure to ultraviolet light.

### Detection of protein synthesis

Oocytes were incubated in M16 medium containing 100 mM L-homopropargylglycine (HPG; a methionine analog that is incorporated into nascent proteins during active protein synthesis) for 1 h and then fixed for 30 min at room temperature in 4% paraformaldehyde (PFA). HPG signals were detected using a Click-iT® HPG Alexa Fluor® Protein Synthesis Assay Kit (Life Technologies). Mean intensity of the HPG signal was measured across the middle of each oocyte and quantified using ImageJ software.

### Cell culture, plasmid transfection and immunoprecipitation

HeLa cells were obtained from ATCC following recent authentication and examination for contamination. Cells were cultured in DMEM (Invitrogen) containing 10% fetal bovine serum (Hyclone) and 1% penicillin-streptomycin solution (Gibco) at 37°C in a humidified 5% CO_2_ incubator. Plasmid transfection was performed using Lipofectamine 2000 (Invitrogen). Cells were lysed in lysis buffer (50 mM Tris-HCl, pH 7.5, 150 mM NaCl, 10% glycerol and 0.5% NP-40) following approximately 48 h of transfection. After centrifugation, the supernatant was incubated with different affinity gels (Sigma-Aldrich). Following incubation at 4°C for 3–4 h, beads were washed with lysis buffer three times. Bead-bound proteins were lysed with sodium dodecyl sulfate (SDS) sample buffer and used for western blotting.

### Quantitative RT-PCR

Total RNA was extracted from oocytes using the RNeasy Mini kit (QIAGEN), according to the manufacturer’s instructions. Next, reverse transcription was performed using the Superscript RT kit (Bio-Rad). Quantitative RT-PCR was performed using a Power SYBR Green PCR Master Mix (Applied Biosystems, Life Technologies) with an ABI 7500 RealTime PCR system (Applied Biosystems), and the primers are listed in Supplementary Table S1.

### Immunofluorescence

Oocytes were fixed with 4% PFA in phosphate-buffered saline (PBS) for 30 min and then permeabilized for 20 min with 0.3% Triton X-100 in PBS. Antibody staining was performed according to previously described standard protocols (29). Antibodies used in the experiments are listed in Supplementary Table S2. Imaging was performed using a Zeiss LSM710 confocal microscope. Semi-quantitative analysis of the fluorescence signals was performed using ImageJ.

### Western blotting

Oocytes were lysed in 2-mercaptoethanol-containing loading buffer and heated at 95°C for 10 min. SDS–polyacrylamide gel electrophoresis and immunoblotting were performed following standard procedures using a Mini-PROTEAN Tetra Cell System (Bio-Rad, Hercules, CA, USA). All antibodies and dilution factors used are listed in Supplementary Table S2.

### Statistical analysis

Results are presented as mean ± standard error of the mean. Most experiments included at least three independent samples and were repeated at least three times. Results for two experimental groups were compared using two-tailed unpaired Student’s *t*-tests; *P < 0.05, **P < 0.01, ***P < 0.001. Results were considered statistically significant at *P* < 0.05. ‘n.s.’ indicates a non-significant result.

## RESULTS

### PAPα plays a key role during oocyte maturation

**Figure 1.**
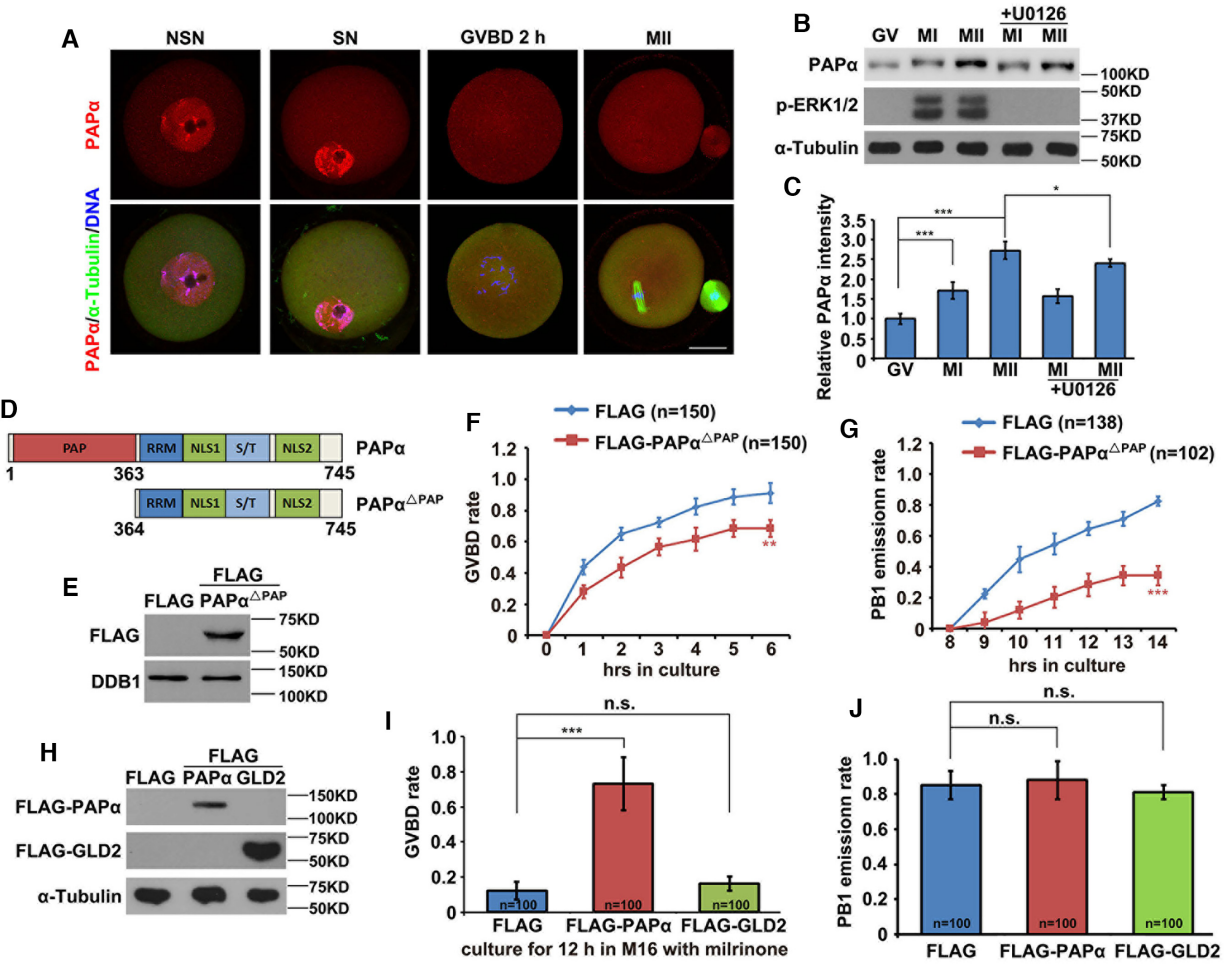
Effect of PAPα and PAPα^△PAP^ overexpression on mouse oocyte meiotic maturation. (**A**) Immunofluorescence results showing localization of endogenous PAPα in GV stage-arrested oocytes having non-surrounded nucleoli (NSN) or surrounded nucleoli (SN), 2 h after GVBD, and in MII oocytes; scale bar: 20 μm. (**B**) Western blotting results showing the protein levels of endogenous PAPα in GV, MI and MII oocytes with or without U0126 treatment. α-Tubulin was used as a loading control; phosphorylated ERK1/2 (p-ERK1/2) was used to indicate meiotic stages and ERK1/2 activation. Total proteins from 200 oocytes were loaded in each lane. (**C**) Quantification of the Western blotting results in (B). (**D**) Schematic representation of human PAPα and PAPα^△PAP^ functional domains. (**E**) Western blotting results showing PAPα^△PAP^ levels in GV oocytes 16 h after microinjection. FLAG was expressed as a negative control; DDB1 was used as a loading control. Control oocytes were microinjected with an *in vitro* transcription product of empty expressing plasmid encoding FLAG. (**F**) Comparison of GVBD kinetics in cultured WT and PAPα^△PAP^-overexpressing oocytes. (**G**) Kinetics of polar body 1 emission (PBE). Oocytes that underwent GVBD within 2 h were selected for further culture. (**H**) Western blotting results showing levels of PAPα and GLD2 in GV oocytes 12 h after microinjection. (**I**) GVBD rates of cultured control, PAPα and GLD2-overexpressing oocytes in M16 medium with 2 μM milrinone. Microinjected GV oocytes were continuously cultured for 12 h in M16 containing milrinone. (**J**) PBE rates of cultured control, PAPα and GLD2-overexpressing oocytes. GV oocytes were first cultured for 8 h in M16 containing milrinone. The oocytes that had undergone GVBD were selected and further cultured for an additional 8 h in milrinone-free M16. PB1 emission rate was measured after culturing. Error bars, standard error of the mean (SEM). *P < 0.05, **P < 0.01, ***P < 0.001.

PAPα proteins are exclusively located in the nucleus of somatic cells (30). Although we found that PAPα was mainly concentrated in the nucleus, it was detected at low levels in the cytoplasm of GV oocytes (Figure 1A and Supplementary Figure S1A); furthermore, it was evenly distributed in the cytoplasm of GVBD and MII oocytes (Figure 1A). Western blotting results showed that the PAPα protein level gradually increased during oocyte meiosis (Figure 1B and C), indicating that PAPα may play a role in cytoplasmic polyadenylation of mRNAs. Next, we knocked down PAPα expression by microinjecting siRNA into GV oocytes. The *Papola* mRNA level was reduced to approximately 10% compared to that in WT (Supplementary Figure S1B). Nevertheless, as PAPα proteins cannot be completely deleted (Supplementary Figure S1C and D), GVBD and polar body emission (PBE) showed minor defects (Supplementary Figure S1E and F).

PAPα contains a catalytic PAP domain at the N-terminus, a predicted RNA recognition motif (RRM), and two C-terminal nuclear localization sequences (NLS1 and NLS2) separated by a Ser/Thr-rich region (Figure 1D) (31,32). To investigate the effects of PAPα on oocyte meiosis, we constructed a dominant negative PAPα mutant without a catalytic PAP domain (PAPα^△PAP^). Although PAPα^△PAP^ failed to catalyze mRNA polyadenylation, it blocked the binding of endogenous PAPα with the CPSF complex and therefore had a dominant negative effect. We expressed FLAG-tagged PAPα^△PAP^ in GV oocytes using mRNA microinjection, and their expression was confirmed by western blotting (Figure 1E). As a result, PAPα^△PAP^ overexpression caused a moderate decrease in the GVBD rate, significantly inhibiting the PBE (Figure 1F and G).

To analyze the effect of WT PAPα overexpression on oocyte meiosis, we expressed FLAG-tagged PAPα in GV oocytes, culturing them for 12 h in a medium containing 2 μM milrinone, which inhibits meiotic resumption. As a negative control, we expressed FLAG-tagged GLD2 (Figure 1H). Although these oocytes were cultured in a medium with milrinone, overexpression of PAPα, instead of GLD2, triggered GVBD (Figure 1I). The oocytes that underwent GVBD within 8 h in the presence of milrinone were selected and further cultured for 8 h in milrinone-free medium. The PB1 emission rate was measured after the culture. Oocytes in each group showed similar PBE rates (Figure 1J). Collectively, this observation suggests that an increased amount and activity of PAPα is necessary and sufficient for normal meiotic cell cycle resumption in fully grown mouse oocytes.

### PAPα is indispensable for normal spindle organization and chromosome alignment in oocyte meiosis

**Figure 2.**
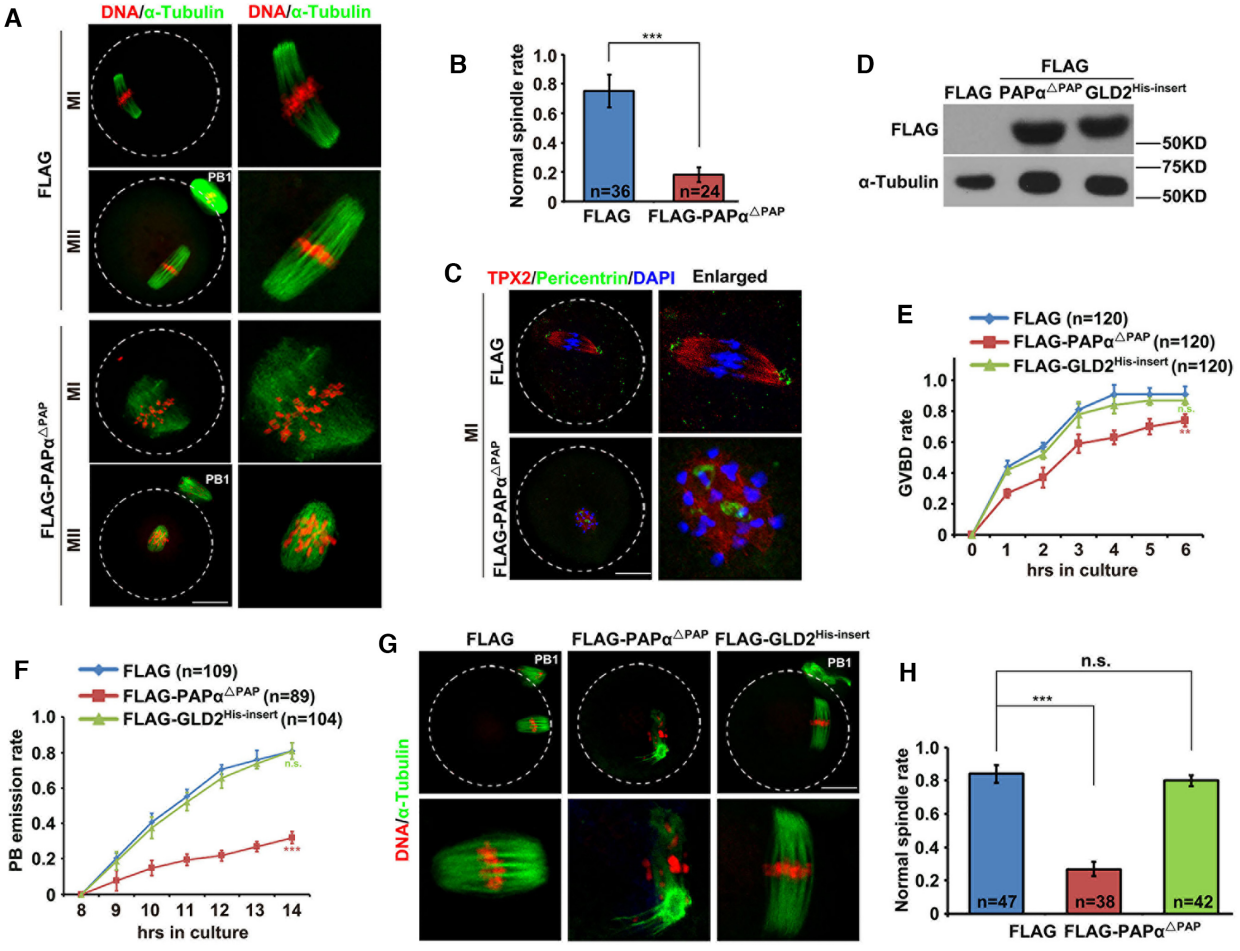
PAPα^△PAP^ overexpression specifically caused defects in oocyte meiosis. (**A**) Results of confocal microscopy showing spindle assembly and chromosome alignment in oocytes microinjected with mRNAs encoding FLAG or FLAG-PAPα^△PAP^. Immunofluorescent images of MI and MII spindles at 8 and 14 h after release from milrinone, respectively; scale bar: 20 μm. (**B**) Rates of oocytes exhibiting normal MII spindle morphology 16 h after mRNA microinjection, as in (A). (**C**) Pericentrin immunofluorescence showing MTOCs in WT and PAPα^△PAP^-overexpressing oocytes at the MI stage. DNA and spindle were labeled using DAPI and microtubule nucleation factor TPX2, respectively; scale bar: 20 μm. (**D**) Western blotting results showing levels of PAPα^△PAP^ and GLD2^His-insert^ in GV oocytes 16 h after microinjection. α-Tubulin was used as a loading control. Total proteins from 60 oocytes were loaded in each lane. (**E**) Comparison of GVBD kinetics in cultured PAPα^△PAP^- and GLD2^His-insert^-overexpressing oocytes. (**F**) Kinetics of polar body emission (PBE). Oocytes that underwent GVBD within 2 h were selected for further culture. (**G**) Results of confocal microscopy showing spindle assembly and chromosome alignment in oocytes microinjected with mRNAs encoding PAPα^△PAP^ and GLD2^His-insert^; scale bar: 20 μm. (**H**) Rates of oocytes exhibiting normal spindle morphology 16 h after mRNA microinjection, as in (**G**). Error bars, standard error of the mean (SEM); **P < 0.01, ***P < 0.001. ‘n.s.’ indicates a non-significant result.

Next, we examined the effect of PAPα^△PAP^ overexpression on spindle assembly. Immunofluorescence results showed that spindle organization and chromosome alignment were remarkably impaired in most MI stage-arrested oocytes, with few developing to MII stage (Figure 2A and B). In WT oocytes, pericentrin, the pivotal component of the microtubule-organizing center (MTOC), was localized in several clusters in the polar area of the MI spindle, but these clusters failed to localize in the polar area in PAPα^△PAP^-overexpressing MI oocytes. This led to disordered distribution of TPX, a microtubule nucleation factor (Figure 2C).

To demonstrate the specificity of the phenotype resulting from PAPα^△PAP^ overexpression, we constructed a dominant negative GLD2 mutant. A previous study showed that insertion of a His residue between the GLD2 amino acids T439 and N440 abolished GLD2 polyadenylation activity (33). Upon GLD2^His-insert^ expression in GV oocytes, GVBD and PBE showed minimal defects (Figure 2D–F), and spindle organization and chromosome alignment were not significantly affected (Figure 2G and H). Taken together, these results indicate that normal PAPα function is indispensable for oocyte meiotic progression.

### Overexpression of dominant negative PAPα caused failure of mRNA poly(A) tail extension and translational activation

**Figure 3.**
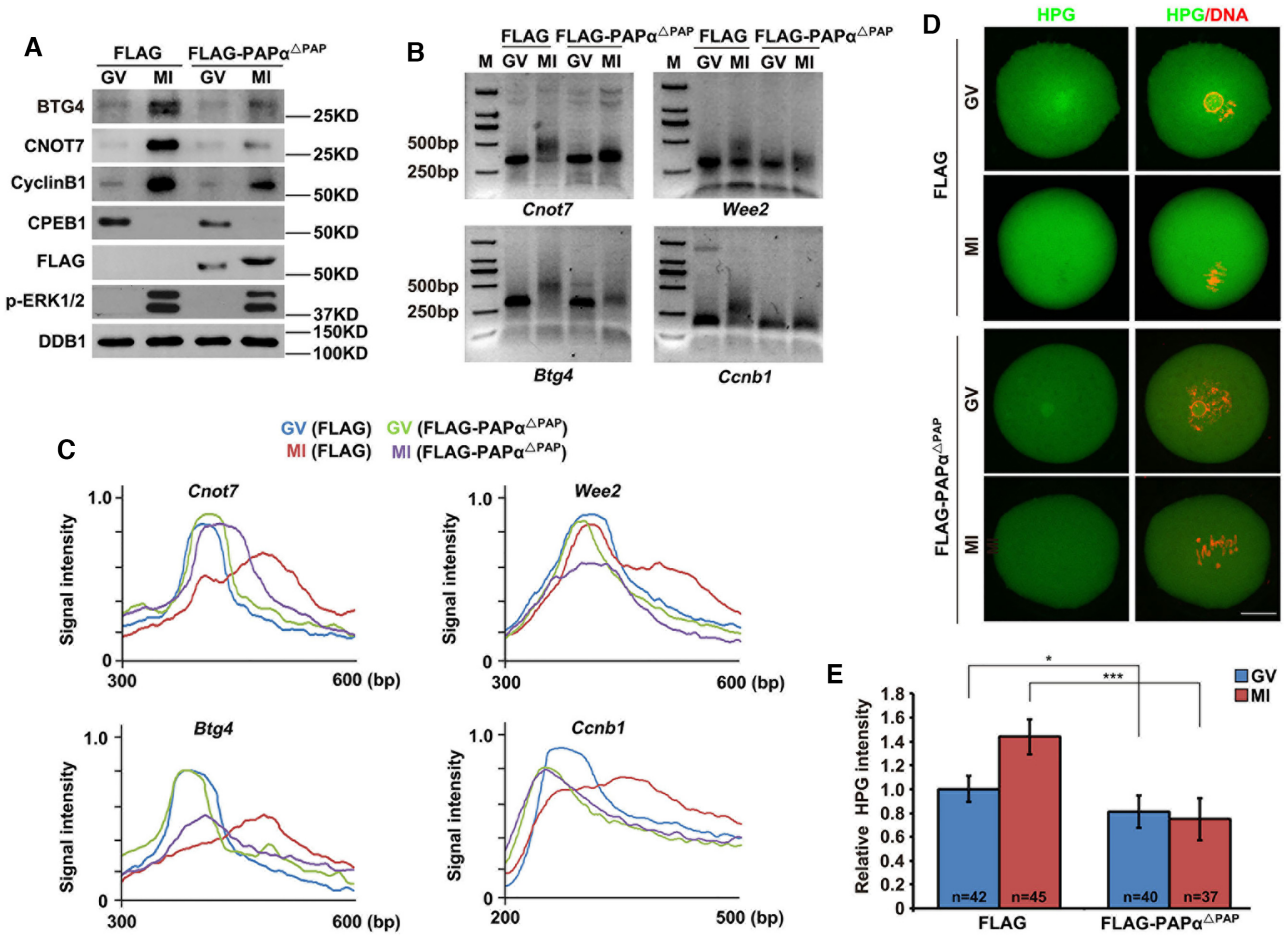
PAPα^△PAP^ overexpression impaired cytoplasmic mRNA polyadenylation and translation in oocytes. (**A**) Western blotting results showing the level of indicated proteins in GV and MI oocytes with or without PAPα^△PAP^ expression. DDB1 was used as a loading control. p-ERK1/2 was used to indicate meiotic stages. Total proteins from 100 oocytes were loaded in each lane. (**B**) PAT assay results showing poly(A) tail lengths of the indicated transcripts in GV and MI oocytes with or without PAPα^△PAP^ expression. (**C**) Quantification of the PAT assay results in (B). The plots show the averaged relative signal intensity (*y*-axis) and the length of the PCR products based on mobility (*x*-axis). (**D**) HPG fluorescent staining results showing protein synthesis activity in GV and MI oocytes with or without PAPα^△PAP^ expression. These oocytes were incubated in a medium containing 50 μM HPG for 1 h before staining; scale bar: 20 μm. (**E**) Quantification of HPG signal intensity in (D). Error bars, standard error of the mean (SEM). *P < 0.05, ***P < 0.001.

Next, we examined the effect of PAPα^△PAP^ overexpression on mRNA polyadenylation and translational activation. Cyclin B1, a key cell cycle-regulated protein, mediates the G2/M transition (34). BTG4 and CNOT7 play important roles in maternal mRNA clearance and maternal-zygotic transition (35). As such, the translational level of mRNAs encoding these proteins should be increased during oocyte maturation; however, these proteins did not accumulate due to PAPα^△PAP^ expression at the MI stage (Figure 3A). In addition, CPEB1, which is highly expressed at the GV stage, showed lower levels than those in the WT (Figure 3A). Next, we examined the polyadenylation level of endogenous transcripts using a PAT assay. Using the PAT assay, previous studies have shown that mRNA polyA tail elongation is most significantly detected at the MI stage, and the bands become weaker at the MII stage owing to meiosis-coupled mRNA degradation (35,36). Because of PAPα^△PAP^ overexpression, extension of the poly(A) tails of *Ccnb1*, *Wee2* (*Wee1* homolog 2), *Btg4* and *Cnot7* was significantly suppressed at the MI stage (Figure 3B and C).

During mouse oocyte maturation, translation of cytoplasmic mRNAs is globally activated (29,37,38). To determine the global translation level, we incubated WT and PAPα^△PAP^-overexpressing oocytes with HPG, a methionine analog that can be detected using the Click-iT cell reaction kit (Life Technologies) (39). In the WT group, stronger HPG signals were emitted by MI-stage oocytes than by GV-stage oocytes, suggesting an increase in the total translational level during oocyte maturation. However, in the PAPα^△PAP^ overexpression group, HPG signals of MI-stage oocytes were comparable to those of GV-stage oocytes (Figure 3D and E), indicating inhibition of total translational activation.

According to a previous study, PAPα contains three highly conserved Asp residues, namely 113, 115 and 167, critical for catalytic activity (Supplementary Figure S2A) (40). In our study, these three Asp residues were mutated to Ala, resulting in the expression of PAPα^3DA^-encoding mRNA in GV oocytes. Consistent with the PAPα^△PAP^ overexpression phenotype, GVBD rates showed a moderate decrease, whereas PB1 emission was significantly impaired (Supplementary Figure S2B and D). Once oocytes resumed meiosis, translational activation of mRNAs encoding cell cycle-related proteins was blocked (Supplementary Figure S2E). Furthermore, significant defects in spindle assembly occurred in MI-arrested oocytes (Supplementary Figure S2F and G). Taken together, these results indicate that PAPα is a key PAP, mediating cytoplasmic mRNA polyadenylation and translation during mouse oocyte maturation.

### CDK1 and ERK1/2 coordinately regulate PAPα phosphorylation in mouse oocytes during meiotic maturation

**Figure 4.**
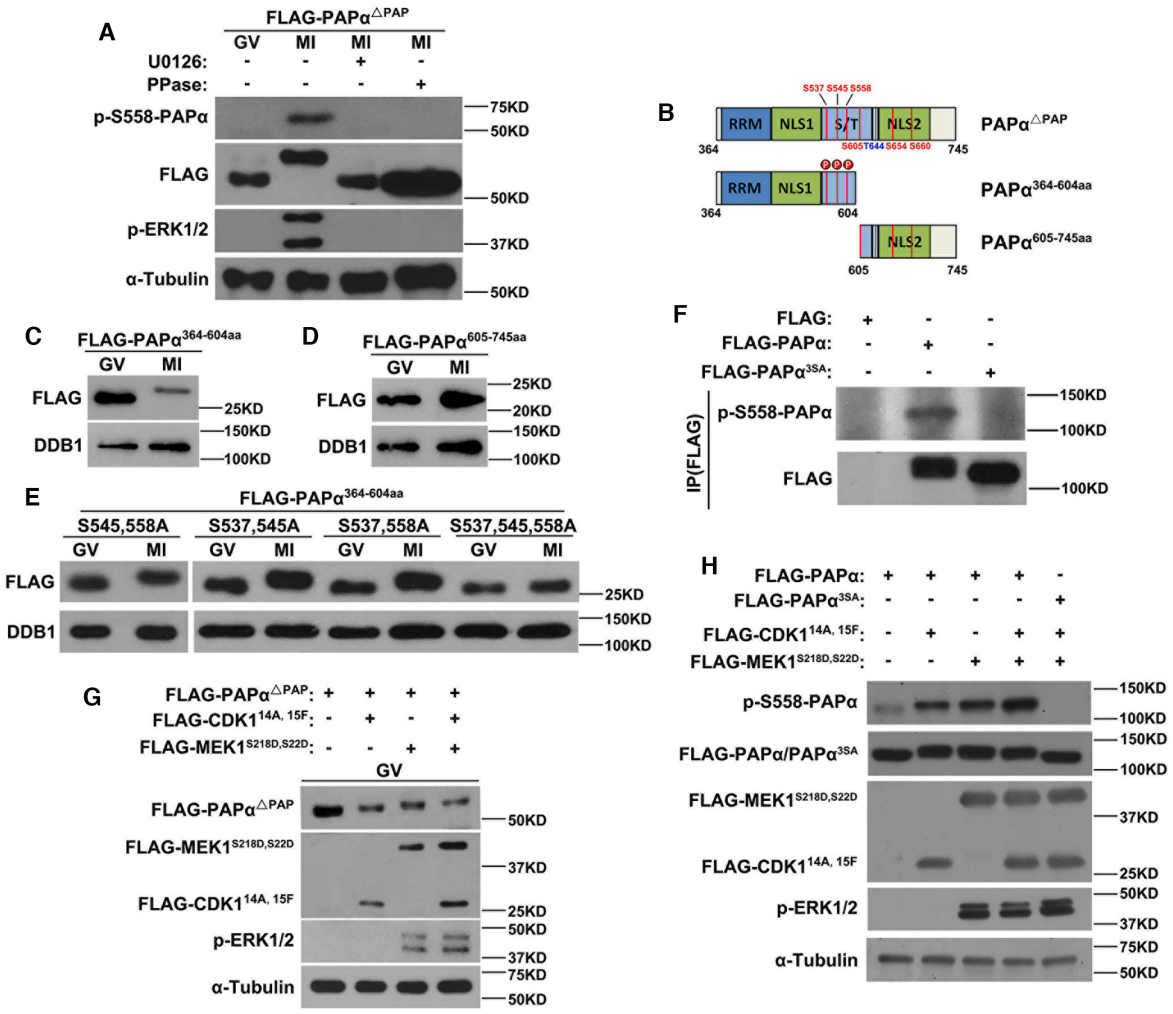
ERK1/2- and CDK1-mediated PAPα phosphorylation during mouse oocyte maturation. (**A**) phospho-S558 PAPα antibody detected the phosphorylation status of PAPα^△PAP^ in GV and MI oocytes with or without U0126 treatment. Some samples were pre-incubated with protein phosphatase for 30 min before western blotting. α-Tubulin was used as a loading control; p-ERK1/2 was used to indicate ERK1/2 activation. Total proteins from 60 oocytes were loaded in each lane. (**B**) The functional domains and potential phosphorylation sites of truncated derivatives of PAPα. (**C** and **D**) PAPα^364–604aa^ instead of PAPα^605–745aa^ was phosphorylated in MI oocytes. (**E**) Western blotting results showing that the mutation of three phosphorylation sites prevented PAPα^364–604aa^ phosphorylation in MI oocytes overexpressing PAPα^364–604aa^. (**F**) Phospho-S558 PAPα antibody detected the phosphorylation status of different forms of PAPα obtained using immunoprecipitation. (**G**) Both CDK1 and ERK1/2 contributed to PAPα phosphorylation in GV oocytes cultured in a medium containing 2 μM milrinone for 12 h. (**H**) Phospho-S558 PAPα antibody detected the phosphorylation status of PAPα in HeLa cells with or without expression of continuously activated CDK1 and MEK1. HeLa cells were transfected with expression plasmids encoding the indicated proteins and were collected for western blotting at 48 h after transfection.

According to our western blotting results, a slight upshift of the PAPα band was observed at the MI and MII stages, which was abolished through the addition of U0126, an inhibitor of ERK1/2 activation, at the start point of *in vitro* maturation culture (Figure 1B), suggesting that endogenous PAPα is phosphorylated in a meiosis-coupled and ERK1/2-dependent manner. ERK1/2 inhibition does not delay GVBD but leads to aberrant meiotic division due to maternal mRNA translation defects (11,41). Additionally, FLAG-tagged PAPα^△PAP^ showed a more significant upshift than the WT PAPα because of its lower molecular weight (Figure 4A), indicating that the modified residue(s) were located outside the PAPα catalytic domain. To examine whether this upshift was caused by phosphorylation modification, we treated the protein sample with phosphatase. As expected, the upshift disappeared. Furthermore, addition of U0126 resulted in the disappearance of this upshift (Figure 4A). Next, by expressing truncated derivatives of PAPα, we identified corresponding phosphorylation sites (Figure 4B). According to a prediction, PAPα contains seven potential phosphorylation sites at the C-terminus. Our results showed that phosphorylation occurred in PAPα^364–604aa^ instead of PAPα^605–745aa^ at the MI stage (Figure 4C and D). As PAPα^364–604aa^ contains three phosphorylation sites, we constructed four mutant forms of PAPα^364–604aa^, namely PAPα^364–604aa (S537,545A)^, PAPα^364–604aa (S537,558A)^, PAPα^364–604aa (S545,558A)^ and PAPα^364–604aa (S537,545,558A)^. However, phosphorylation was only prevented when the three sites (S537, S545 and S558) were simultaneously mutated (Figure 4E), indicating that these sites all contribute to PAPα phosphorylation. Using a self-generated phospho-PAPα-S558 antibody, we further confirmed the phosphorylation of PAPα during oocyte maturation, which is regulated by ERK1/2 (Figure 4A). In addition, we demonstrated the specificity of the phospho-PAPα-S558 antibody by expressing WT PAPα and PAPα^3SA^ in HeLa cells (Figure 4F). Unfortunately, this antibody failed to detect the endogenous phosphorylated PAPα, probably because of low PAPα expression and low antibody sensitivity.

During mouse oocyte maturation, maturation promoting factor (a heterodimer of CDK1 and cyclin B) and MAPK, two key kinase cascade molecules, regulate the phosphorylation of a series of cell cycle-related proteins (12,42). As PAPα can be phosphorylated by CDK1 and ERK1/2 in somatic cells (18,43), we examined whether they contribute to PAPα phosphorylation during oocyte meiosis. As a CDK1 inhibitor, roscovitine causes the failure of oocyte GVBD; herein, we expressed continuously activated CDK1 and MEK1 (CDK1^T14A,Y15F^ and MEK1^S218D,S222D^) in GV oocytes and collected these oocytes for western blotting. The results showed that CDK1 ^T14A,Y15F^ or MEK1^S218D,S222D^ alone could trigger PAPα phosphorylation. Notably, overexpression of CDK1 ^T14A,Y15F^ and MEK1^S218D,S222D^ together led to further phosphorylation (Figure 4G), indicating their coordinated regulation of PAPα phosphorylation during mouse oocyte maturation. Furthermore, we directly detected the phosphorylation status of the PAPα S558 site in HeLa cells overexpressing constitutively active CDK1 and MEK, using the phosphorylation-specific antibody we generated. The level of S558-phosphorylated PAPα was significantly increased in cells expressing CDK1 and/or MEK1 (Figure 4H). A FLAG antibody also detected an upshift of FLAG-PAPα bands in the presence of CDK1 and/or MEK1. As a negative control, the phosphorylation site mutated PAPα (PAPα^3SA^) were not detected by the anti-pPAPα-S558 antibody in the presence of CDK1 and MEK1. Nor did PAPα^3SA^ upshifted when CDK1 and MEK1 were co-expressed (Figure 4H).

### RRM of PAPα is responsible for interactions between PAPα and the CPSF complex

**Figure 5.**
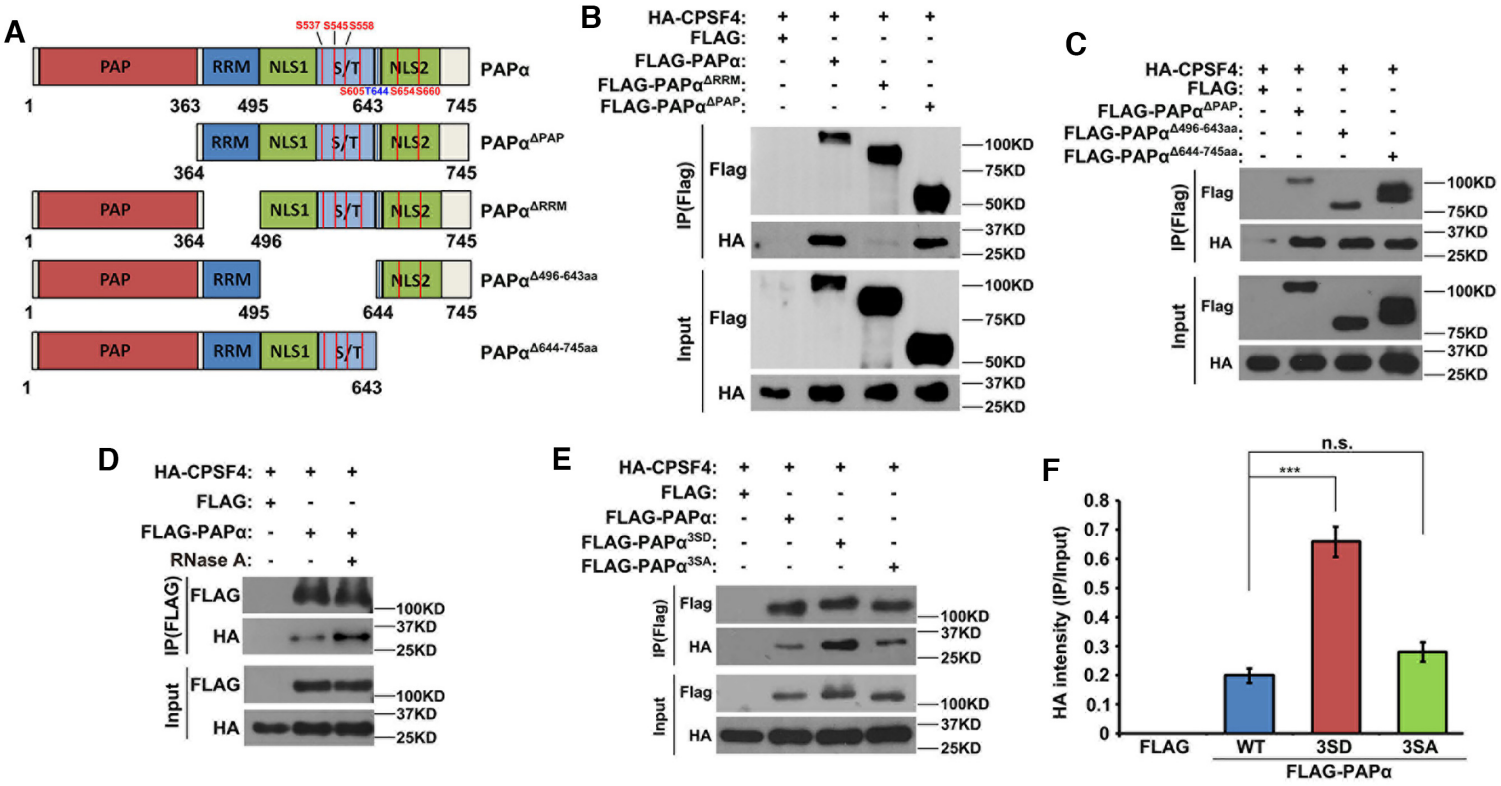
RRM contributed to the binding between PAPα and the CPSF complex. (**A**) Schematic representation of the functional domains of PAPα and truncated derivatives of PAPα. (**B**) Co-IP assay showing the binding of CPSF4 with PAPα, PAPα^△PAP^ and PAPα^△RRM^ in HeLa cells transfected with plasmids expressing the indicated proteins. (**C**) Co-IP assay showing the binding of CPSF4 with PAPα, PAPα^△496–643aa^ and PAPα^△644–745aa^. (**D**) Co-IP assay showing the binding of CPSF4 with PAPα with or without RNase A treatment. (**E**) Western blotting after Co-IP assay showing the binding of CPSF4 with different phosphorylated forms of PAPα. (**F**) Relative level of HA-CPSF4 when comparing immunoprecipitation and input in (E). Error bars, standard error of the mean (SEM); ***P < 0.001, ‘n.s.’ indicates a non-significant result.

According to a previous study, although PAPα possesses an RRM, it has extremely low affinity for RNA substrates (44). PAPα is recruited to pre-mRNA, in part, by CPSF complex binding (45), for which the responsible domain is not yet known. To investigate this, we constructed several truncated derivatives of PAPα, namely PAPα^△PAP^, PAPα^△RRM^, PAPα^△496–643aa^ and PAPα^△644–745aa^ (Figure 5A). HA-tagged CPSF4 and different truncated derivatives of PAPα were co-expressed in HeLa cells. Of the four PAPα derivatives, only PAPα^△RRM^ abolished CPSF complex binding (Figure 5B and C). These results indicate that the interactions between PAPα and the CPSF complex may rely on mRNA binding. We then treated HeLa cell lysates containing exogenously expressed PAPα and CPSF4 with RNase A. However, RNase A treatment had a minor influence on the binding of PAPα to the CPSF complex (Figure 5D). Collectively, although these results suggest that the predicted RRM of PAPα is indispensable for CPSF binding; they interact with each other in an RNA-independent manner.

Next, we examined the effect of PAPα phosphorylation on the interaction between PAPα and the CPSF complex. We co-expressed HA-tagged CPSF4 and different mutant forms of FLAG-tagged PAPα in HeLa cells. Although IP results showed that PAPα interacted with the CPSF complex, phosphorylation-mimicking PAPα (PAPα^3SD^) showed a stronger binding affinity than PAPα^WT^ toward the CPSF complex, while PAPα^3SA^ and PAPα^WT^ displayed comparable affinity for the CPSF complex (Figure 5E and F). These results suggested that phosphorylation promotes PAPα polyadenylation activity by increasing the interaction between PAPα and the CPSF complex. However, as these co-IP assays did not provide a quantitative measurement for the affinity, per se, the biochemical role of PAPα phosphorylation requires further investigation.

### Phosphorylation promotes PAPα polyadenylation activity in maturing mouse oocytes

**Figure 6.**
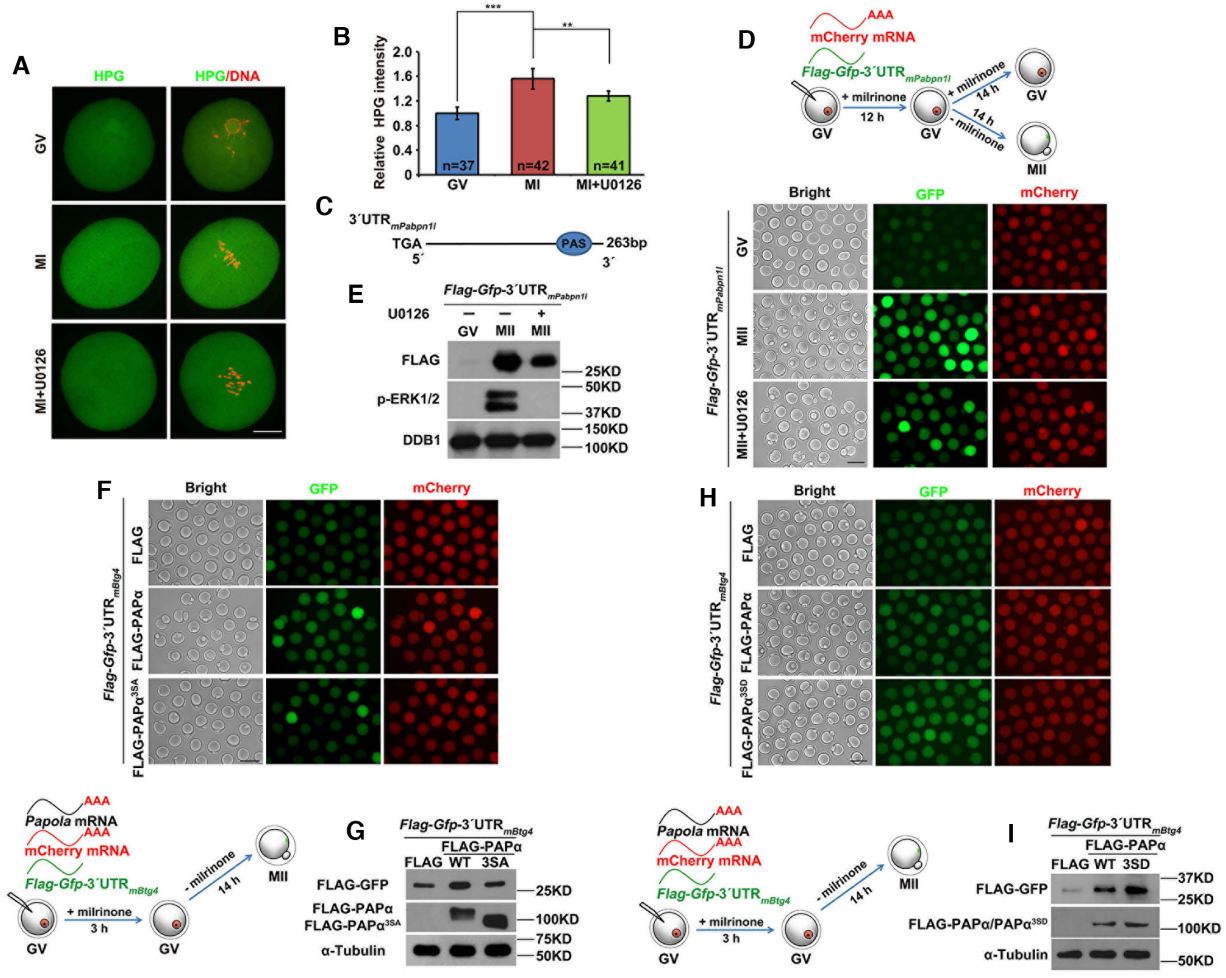
Contribution of phosphorylation to PAPα activity during oocyte meiosis. (**A**) HPG fluorescent staining results showing protein synthesis activity in GV and MI oocytes with or without U0126 treatment. These oocytes were incubated in a medium containing 50 μM HPG for 1 h prior to staining; scale bar: 20 μm. (**B**) Quantification of HPG signal intensity in (A). (**C**) Schematic representation of the 3′-UTR of mouse *Pabpn1l* mRNA. (**D** and **E**) Fluorescence microscopy (D) and western blotting (E) results showing *Pabpn1l* 3′-UTR expression in GV and MII oocytes with or without U0126 treatment. DDB1 was used as a loading control. Total proteins from 60 oocytes were loaded in each lane; scale bar: 100 μm. (**F** and **G**) Fluorescence microscopy (F) and western blotting (G) results showing the expression of PAPα, phosphorylation inactivation mutant of PAPα, and FLAG-GFP fused with *Btg4* 3′-UTR. (**H** and **I**) Fluorescence microscopy (H) and western blotting (I) results showing the expression of PAPα, phosphorylation activation mutant of PAPα, and FLAG-GFP fused with *Btg4* 3′-UTR. Error bars, standard error of the mean (SEM); **P < 0.01, ***P < 0.001.

HPG fluorescent staining results indicated that the global mRNA translation level was elevated once oocytes resumed meiosis (29); the addition of U0126 weakened translational activation (Figure 6A and B). Although previous studies have primarily attributed translational activation to CPEB1-dependent regulation (16,46), it occurs in many transcripts with the 3′-UTR lacking CPEs. An example of this is *Pabpn1l*, which encodes nuclear poly(A) binding protein 1 like (PABPN1L), a key maternal-zygotic transition factor (47). The 3′-UTR of mouse *Pabpn1l* (3′-UTR_m_*_Pabpn1l_*) only contains a PAS without adjacent CPEs (Figure 6C). After cloning 3′-UTR_m_*_Pabpn1l_* and inserting it into a pRK5-*Flag*-*Gfp* vector, we injected un-polyadenylated mRNA encoding *Flag*-*Gfp*-3′-UTR_m_*_Pabpn1l_* into GV oocytes. As a control for background translational activity, an *in vitro*-polyadenylated mRNA encoding mCherry cDNA was co-injected. Although the 3′-UTR_m_*_Pabpn1l_* lacks CPE, GFP fluorescence and FLAG western blotting results showed significant translational activation during oocyte maturation. Furthermore, the addition of U0126 partially impaired translational activation (Figure 6D and E). These results suggested the existence of a CPEB1-independent mechanism of meiotic cell cycle-coupled translational activation in mouse oocytes.

**Figure 7.**
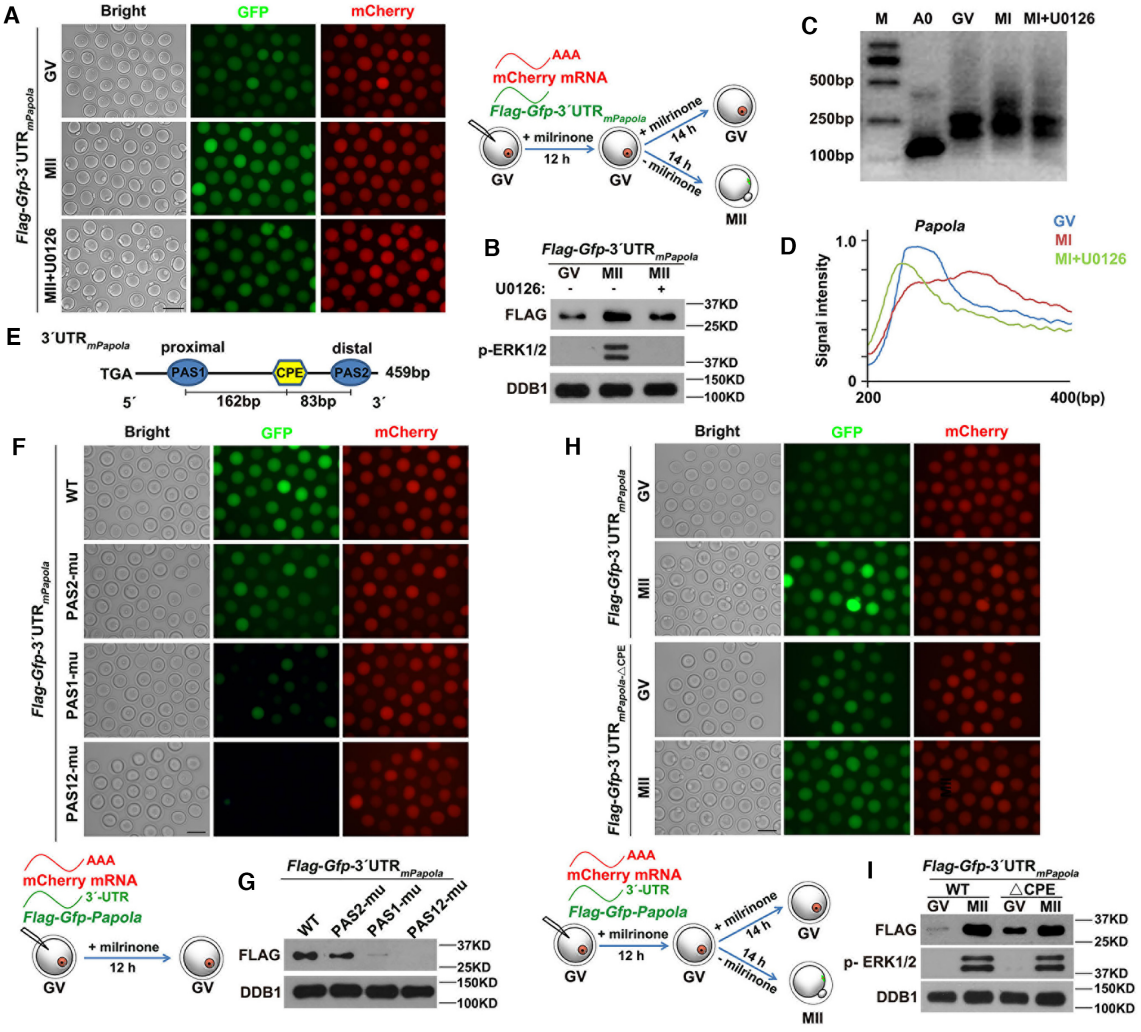
PAPα regulates its own translation through a positive feedback circuit. (**A** and **B**) Fluorescence microscopy (A) and western blotting (B) results showing the expression of FLAG-GFP fused with mouse *Papola* 3′-UTR in GV and MII oocytes with or without U0126 treatment. DDB1 was used as a loading control, and phosphorylated ERK1/2 (p-ERK1/2) was used to indicate the cell cycle stage and ERK1/2 activation. Total protein from 60 oocytes was loaded into each lane; scale bar: 100 μm. (**C**) Poly(A) tail assay results showing changes in the poly(A) tail length of *Papola* mRNA in GV and MI oocytes with or without U0126 treatment. (**D**) Quantification of the PAT assay results in (C). The plots showed the averaged relative signal intensity (*y*-axis) and the length of the PCR products based on mobility (*x*-axis). (**E**) Schematic representation of the 3′-UTR of *Papola* mRNA; relative positions of PAS and CPE are indicated. (**F** and **G**) Fluorescence microscopy (F) and western blotting (G) results showing the translation levels of FLAG-GFP driven by *Papola* 3′-UTR or its PAS-mutated (△PAS) forms in GV oocytes. (**H** and **I**) Fluorescence microscopy (H) and western blotting (I) results showing the translation levels of FLAG-GFP driven by *Papola* 3′-UTRs with or without CPE mutations in GV and MII oocytes.

Based on the above results, we speculated that phosphorylation enhances PAPα activity by promoting its binding to the CPSF complex, leading to cytoplasmic poly(A) tail elongation and translational activation of a broad spectrum of maternal mRNAs. To confirm our hypothesis, we cloned the 3′-UTR of mouse *Btg4* (3′-UTR_m_*_Btg4_*) and inserted it into a pRK5-*Flag*-*Gfp* vector. PAPα polyadenylation activity was traced by comparing 3′-UTR_m_*_Btg4_*translation level changes. Next, we co-injected different forms of *in vitro*-polyadenylated *Papola* mRNA and un-polyadenylated mRNA encoding *Flag*-*Gfp*-3′-UTR_m_*_Btg4_* into GV oocytes; *in vitro*-polyadenylated mRNA encoding mCherry cDNA was also co-injected. Microinjected oocytes were further cultured for 14 h in M16 medium without milrinone; these MII oocytes were collected for use in subsequent experiments. GFP fluorescence and FLAG western blotting results indicated that PAPα overexpression promoted 3′-UTR_m_*_Btg4_* translation. The positive effect of PAPα overexpression on 3′-UTR_m_*_Btg4_* translation was abolished when three Ser phosphorylation sites were mutated to Ala (PAPα^3SA^) (Figure 6F and G). In contrast, when three Ser residues were mutated to Asp (PAPα^3SD^), which mimicked phosphorylation, the 3′-UTR_m_*_Btg4_*translation level was remarkably more enhanced than that by WT PAPα expression (Figure 6H and I). These results confirmed that the polyadenylation activity of PAPα was elevated through phosphorylation, leading to further polyadenylation and translational activation of global mRNA. Supplementary Figure S3A–F compared the GFP and mCherry fluorescence intensities to quantify the translational activity of the 3′-UTR reporters (Figures 6 and 7). Supplementary Figure S3G-L quantified the intensities of FLAG-GFP bands normalized by the α-tubulin bands detected by western blotting in the same samples (Figures 6 and 7).

### PAPα reinforced its own mRNA polyadenylation and translation through a positive feedback circuit

According to an endogenous expression pattern, PAPα levels gradually increased during oocyte meiosis, while U0126 impaired PAPα accumulation (Figure 1B and C). To further investigate the potential translational regulation of *Papola*, which encodes PAPα, during oocyte maturation, we cloned mouse *Papola* 3′-UTR (3′-UTR_m_*_Papola_*) and inserted it into a pRK5-*Flag*-*Gfp* vector. Consistent with an endogenous expression pattern, the translational activity of 3′-UTR_m_*_Papola_*significantly increased from the GV to the MII stage, with U0126 blocking translation (Figure 7A and B). Next, we detected the polyadenylation level of endogenous *Papola* mRNA using PAT assay. *Papola* mRNA polyadenylation was maintained at a relatively low level in GV oocytes. Although, following GVBD, the poly(A) tail of the *Papola* mRNA was significantly extended, this extension was impaired following U0126 treatment at the MI stage (Figure 7C and D). Taken together, these results suggested that *Papola* mRNA promotes its own polyadenylation and translational activation, with ERK1/2 playing a vital role in the regulation of *Papola* mRNA polyadenylation and translation.

PAS and CPE are the most fundamental elements of mRNA polyadenylation. A previous study revealed a combinatorial code for mRNA 3′-UTR-regulated translational control (21). To further test whether the conclusion is widely applicable, we examined the effects of these sequence elements on *Papola* mRNA polyadenylation and translation. The 3′-UTR_m_*_Papola_*contained a CPE flanked by two PASs (Figure 7E). First, we investigated the contribution of an individual PAS to the translational activity of 3′-UTR_m_*_Papola_* at the GV stage. While mutations in PAS2 only caused minor translational repression, mutations in PAS1 resulted in significant repression. When both PASs were mutated, the 3′-UTR_m_*_Papola_* could not be translated (Figure 7F and G). In contrast, a CPE mutation in 3′-UTR_m_*_Papola_* led to an obvious increase and a moderate decrease in translational activity at the GV and MII stages, respectively (Figure 7H and I). This result indicated that CPEB1 negatively influences *Papola* mRNA translation in GV oocytes via CPE in the *Papola* mRNA 3′-UTR and maintains a low PAPα protein level before meiotic resumption. Presumably, this inhibitory effect is relieved by CDK1/ERK1/2-mediated CPEB1 phosphorylation and degradation during GVBD (11,12). Taken together, these results are consistent with previous conclusions (21): (i) mRNA translation at the GV stage requires one or more PASs distantly located from CPEs; (ii) PAS does not need to locate to the 3′-end of the mRNA to mediate translational activation; and (iii) CPE plays a dual role in the translational regulation of mRNA, impeding and promoting mRNA translation by influencing its adjacent PAS (in this case, the PAS2 of mouse *Papola* 3′-UTR) at the GV and MII stages, respectively.

## DISCUSSION

During oocyte meiosis, maternal mRNAs are combined with short poly(A) tails by nuclear PAPs. These mRNAs are then transported to the cytoplasm to provide material and energy for subsequent meiotic processes (48). Once oocytes resume meiosis, maternal mRNAs with short poly(A) tails are further polyadenylated by cytoplasmic PAPs, resulting in global translational activation (49); however, the key cytoplasmic PAPs during mouse oocyte maturation have not yet been identified. Although GLD2 is recognized as the cytoplasmic PAP in *Xenopus* oocyte meiosis, the phenotype associated with *Gld2*-knockout mice indicates its redundancy in mouse oocytes (23,24). Our study indicated that a nuclear PAP, PAPα, is primarily responsible for the cytoplasmic PAP activity during mouse oocyte maturation. When the dominant negative form of PAPα was expressed in GV oocytes, the normal meiotic process was disrupted, and the polyadenylation and translational activation of cell cycle-related transcripts was inhibited. PAPα was detected not only in the nucleus of GV stage-arrested oocytes but also in the ooplasm. In addition, it is conceivable that nuclear PAPα was released into the ooplasm after GVBD and triggered the cytoplasmic polyadenylation of oocyte transcripts.

To support the dramatically increased cytoplasmic polyadenylation and translation of maternal transcripts during oocyte maturation, regulation of PAPα activity is coupled with meiotic cell cycle resumption. CDK1 and ERK1/2 are key kinases that regulate oocyte meiotic divisions (41,50). We found that CDK1 and ERK1/2 synergistically mediated PAPα phosphorylation. Corresponding phosphorylation sites consisted of three Ser residues, namely 537, 545 and 558, with phosphorylation resulting in a stronger interaction between PAPα and the CPSF complex, thereby promoting PAPα activity. Although it contains a predicted RRM, a previous study has shown that PAPα has low affinity for mRNA. We found that RRM was indispensable for the interaction between PAPα and the CPSF complex, and RNase A treatment failed to block this interaction. These observations suggested that the previously designated RRM in PAPα is actually a CPSF-binding domain. According to published results (21), CPSF binds with the PAS elements in mRNA 3′-UTR and mediates polyadenylation by recruiting canonical PAPs. Blocking CPSF function in mouse oocytes leads to impaired meiotic spindle assembly and PB1 emission. These phenotypes resemble those observed following blockade of PAPα in the current study. These results indicate that the interaction between PAPα and CPSF has a significant role in oocyte meiotic maturation.

**Figure 8.**
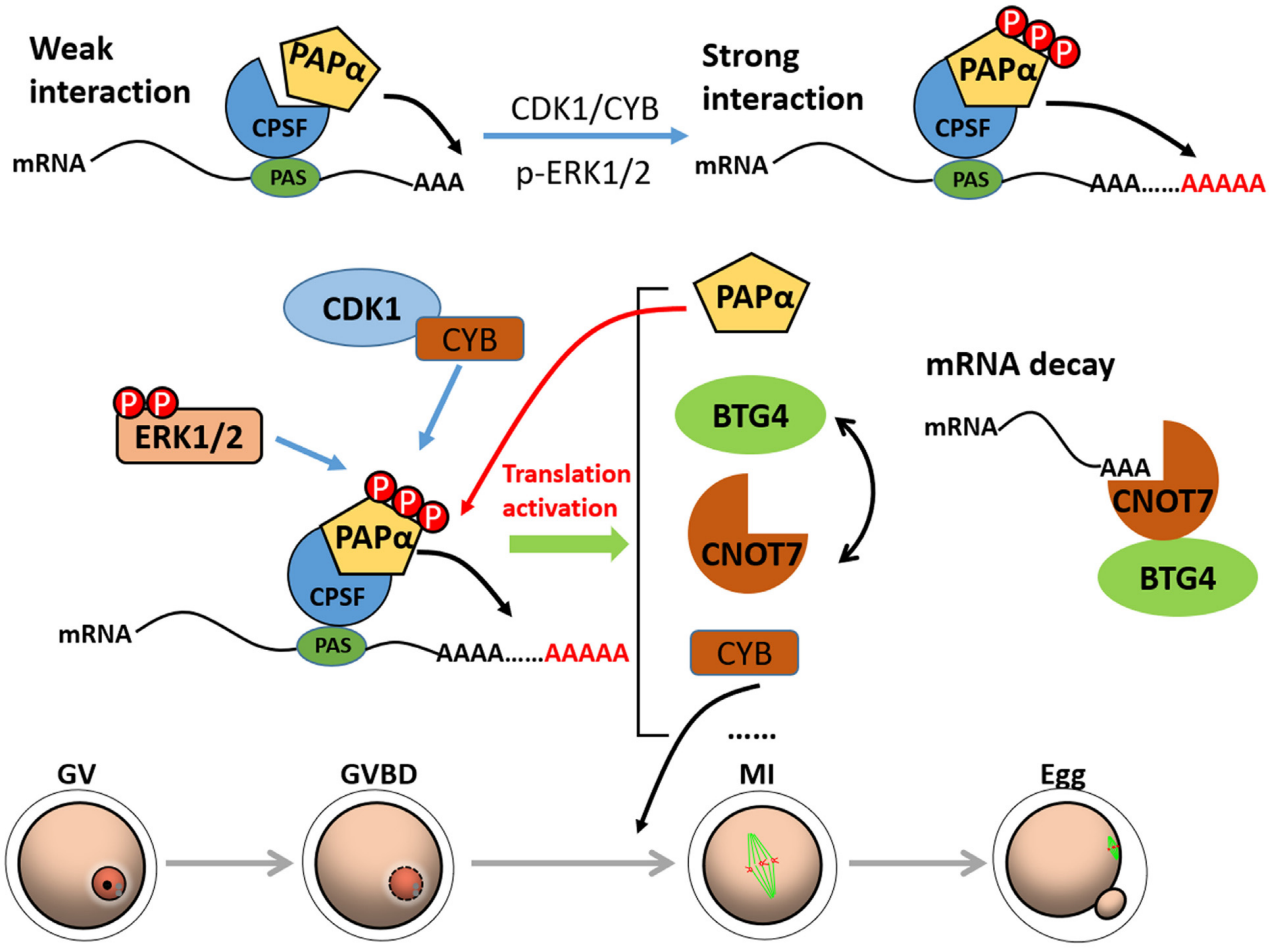
Model representing the regulation of PAPα activity and translation in oocytes. Once oocytes resume meiosis, activated CDK1 and ERK1/2 cause PAPα phosphorylation. Phosphorylated PAPα exhibits stronger binding with the CPSF complex, leading to higher polyadenylation activity. In turn, accumulated and activated PAPα stimulates the polyadenylation of *Papola* mRNA through a positive feedback approach. High catalytic activity and protein levels of PAPα allow the poly(A) tails of cell cycle-related mRNAs stored in the cytoplasm, such as *Cnot7*, *Btg4* and *Ccnb1*, to be further extended, resulting in global translational activation during mouse oocyte maturation.

In addition, our study showed that PAPα levels were elevated via positive feedback regulation and ERK1/2-mediated translational activation of *Papola* transcripts (Figure 8). This positive feedback circuit may play an important role in maintaining PAPα levels. Therefore, PAPα-mediated cytoplasmic polyadenylation is strengthened at least at three levels: (i) release of nuclear PAPα into the ooplasm after GVBD; (ii) increase of PAPα affinity for CPSF by CDK1- and ERK1/2-mediated phosphorylation; and (iii) positive feedback activation of *Papola* mRNA translation during meiotic cell cycle progression. Notably, these mechanisms contribute to a global increase in the cytoplasmic mRNA polyadenylation activities, regardless of the 3′-UTR cis-elements in the target transcripts.

However, in our study, meiotic resumption of fully grown mouse oocytes is only partially inhibited following overexpression of the dominant negative PAPα. This may be because of technical limitations such as the endogenous PAPα not being completely inhibited or the presence of other cytoplasmic PAPs capable of partially substituting the function of PAPα. There is an initial trigger of maternal mRNA translation at the very early stage of oocyte meiotic resumption, before GVBD. The initial accumulation of cyclin B1 and B2, as well as MOS, the upstream kinase of the MAPK cascade, drive the GV stage-arrested oocytes to pass a threshold of cell cycle resumption (12,51–53). However, the role of PAPα in this initial, and relatively subtle, polyadenylation event was not determined in this study, primarily because the assays employed are not sufficiently sensitive and cannot be strictly controlled according to time or developmental stages.

Nevertheless, an interesting result was that PAP overexpression in GV oocytes overcame milrinone blockade. Naturally, PAPα is released to the cytoplasm post GVBD to catalyze polyadenylation. Moreover, CDK1/ERK activity is low/absent in GV oocytes. Although PAPα is primarily concentrated in GV, a substantial fraction is localized in the cytoplasm (Supplementary Figure S1A). In addition, the *in vitro* transcribed and pre-polyadenylated mRNAs encoding PAPα were microinjected into the ooplasm. These mRNAs were immediately translated into PAPα proteins, which may have sufficient time to catalyze polyadenylation of maternal transcripts in the ooplasm before the exogenous PAPα translocates to the GV. In addition, CDK1/ERK-mediated phosphorylation enhances, but is not absolutely required, for the interaction between PAPα and CPSF (Figure 5E). Therefore, the overexpressed unphosphorylated PAPα retains its ability to induce mRNA polyadenylation in GV oocytes, albeit at a lower level. However, the PAPα-overexpressing oocytes did not show advanced PB1 emission, likely because the timing of the MI-MII transition is primarily determined by anaphase-promoting complex-mediated protein polyubiquitination and degradation processes, which is not closely regulated by PAPα (6).

Taken together, these findings have implications on previous theories that attribute translational activation to CPEB1 regulation, as translational activation still occurs in many transcripts in which the 3′-UTR lacks CPEs. Our study explains how global translational activation is regulated during oocyte maturation. Through a variety of signal regulation pathways, PAPα activity and levels are significantly promoted, contributing to subsequent translational activation during oocyte meiosis.

## Supplementary Material

gkab431_Supplemental_File
