# Supplementary material for: Oocyte meiosis-coupled poly(A) polymerase α phosphorylation and activation trigger maternal mRNA translation in mice
Source: Nucleic Acids Res. 2021 May 28;49(10):5867–80. doi: 10.1093/nar/gkab431 (PMC8191758; doi:10.1093/nar/gkab431)
Supplement: gkab431_Supplemental_File [file gkab431_supplemental_file.pdf]

Supplementary Information

Supplementary Figures

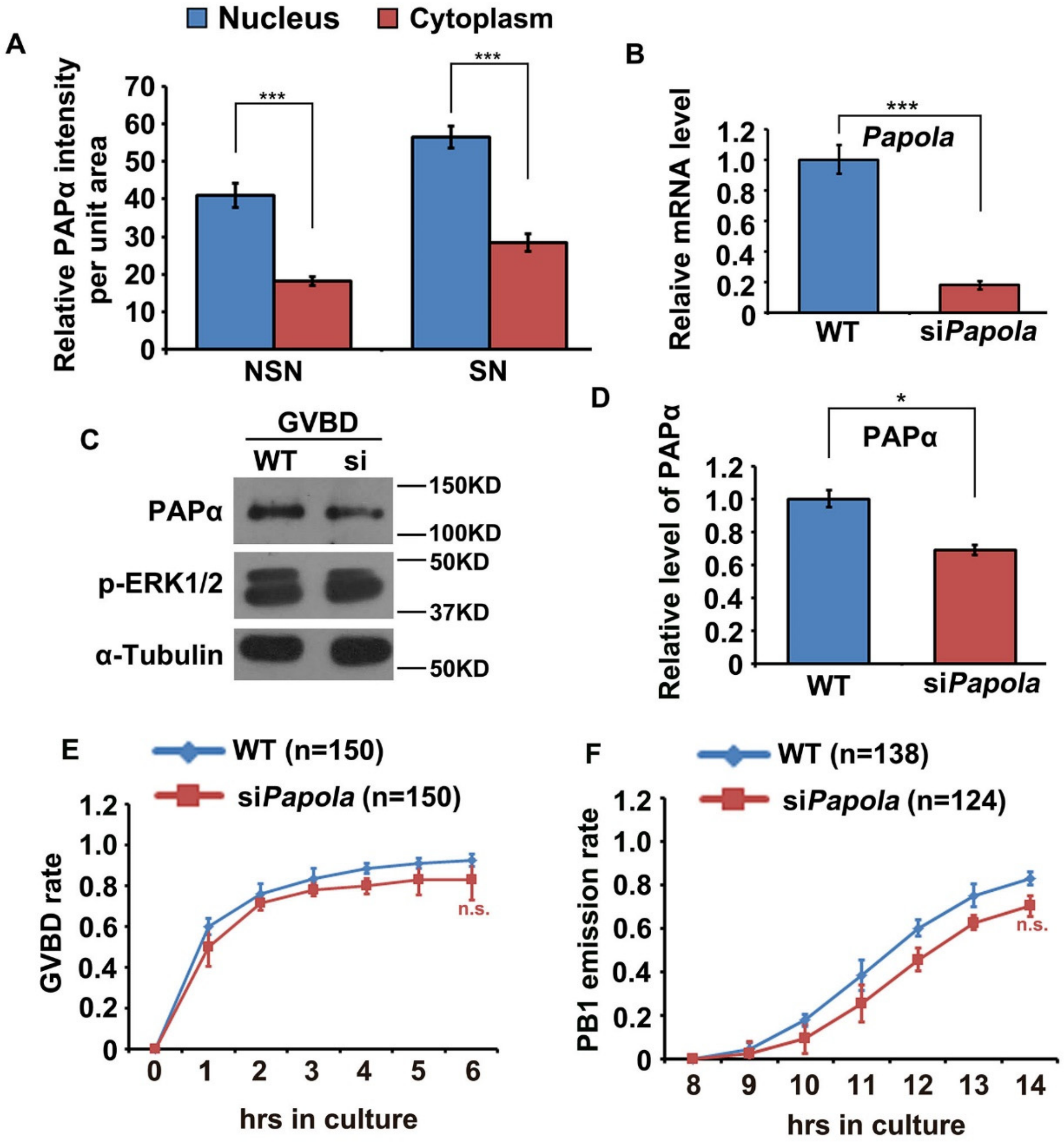

Supplementary Figure S1: The effect of si*Papola* on oocyte meiosis. A: Quantification of the endogenous PAPα protein levels detected by immunofluorescence in nucleus and cytoplasm of NSN and SN oocytes. **B-D:** Quantitative RT-PCR (**B**) and western blotting (**C-D**) results showing the deletion effect of *Papola* siRNA. α-Tubulin was used as a loading control. p-ERK1/2 was used to indicate the meiotic stages. **E:** Comparison of GVBD kinetics in cultured WT and si*Papola*-transfected oocytes. **F:** Kinetics of polar body emission (PBE). Oocytes that underwent GVBD within 2 h were selected for further culture. Error bars, standard error of the mean (SEM). ***$P < 0.001$. “n.s.” indicates a non-significant result.

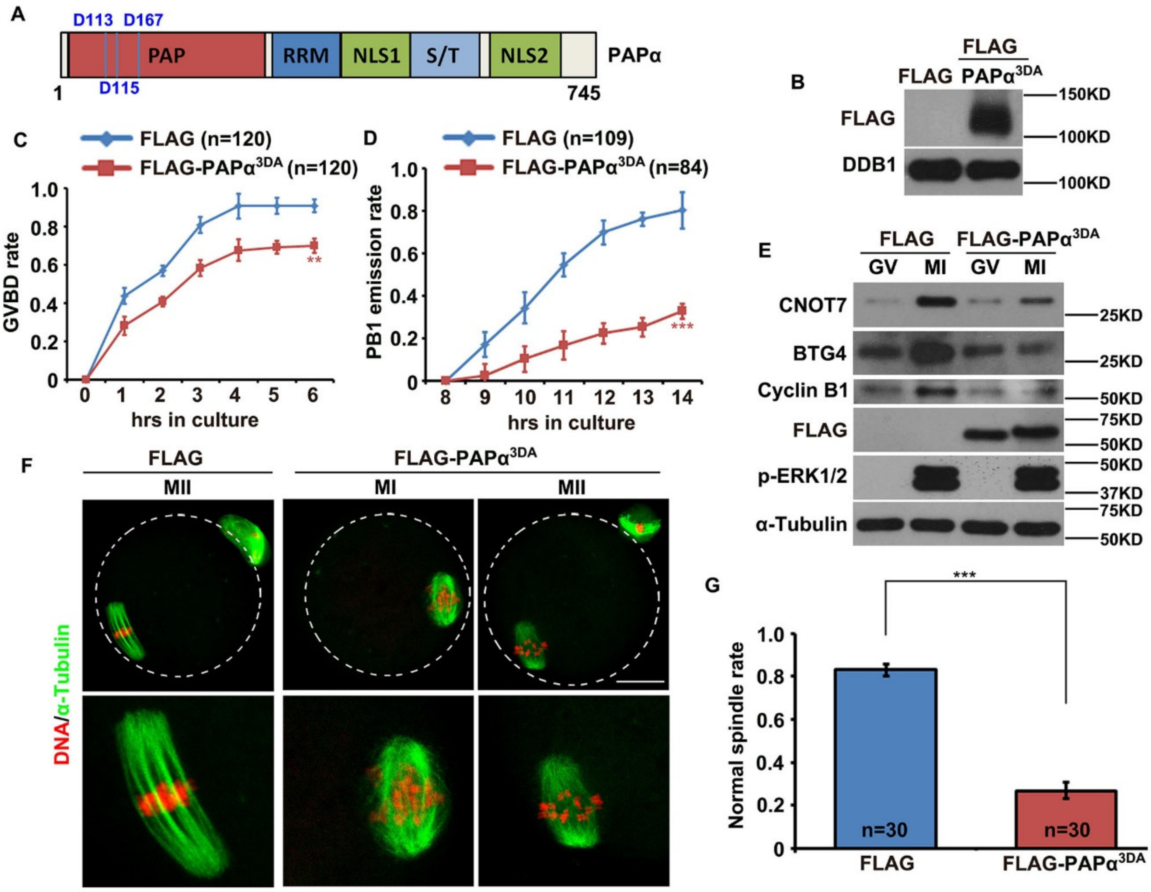

Supplementary Figure S2: PAPα^{3DA} overexpression resulted in a similar phenotype to PAPα$^{\triangle PAP}$ overexpression. A: Schematic representation of three catalytic Asp residues of human PAPα. **B:** Western blotting results showing the expression levels of PAPα^{3DA} in GV oocytes 16 h after microinjection. FLAG was expressed as a negative control, while DDB1 was used as a loading control. Total proteins from 60 oocytes were loaded into each lane. **C:** Comparison of GVBD kinetics in cultured WT and PAPα^{3DA}-overexpressing oocytes. **D:** Kinetics of polar body emission (PBE). Oocytes that underwent GVBD within 2 h were selected for further culture. **E:** Western blotting results showing levels of indicated proteins in GV and MI oocytes with or without PAPα^{3DA} expression. α-Tubulin was used as a loading control. Total proteins from 100 oocytes were loaded into each lane. **F:** Results of confocal microscopy showing spindle assembly and chromosome alignment in oocytes microinjected with mRNAs encoding FLAG and PAPα^{3DA}. Scale bar, 20 µm. **G:** Rates of oocytes exhibiting normal spindle morphology 16 h after mRNA microinjection, as in F. Error bars, standard error of the mean (SEM). $^{**}P < 0.01$, $^{***}P < 0.001$.

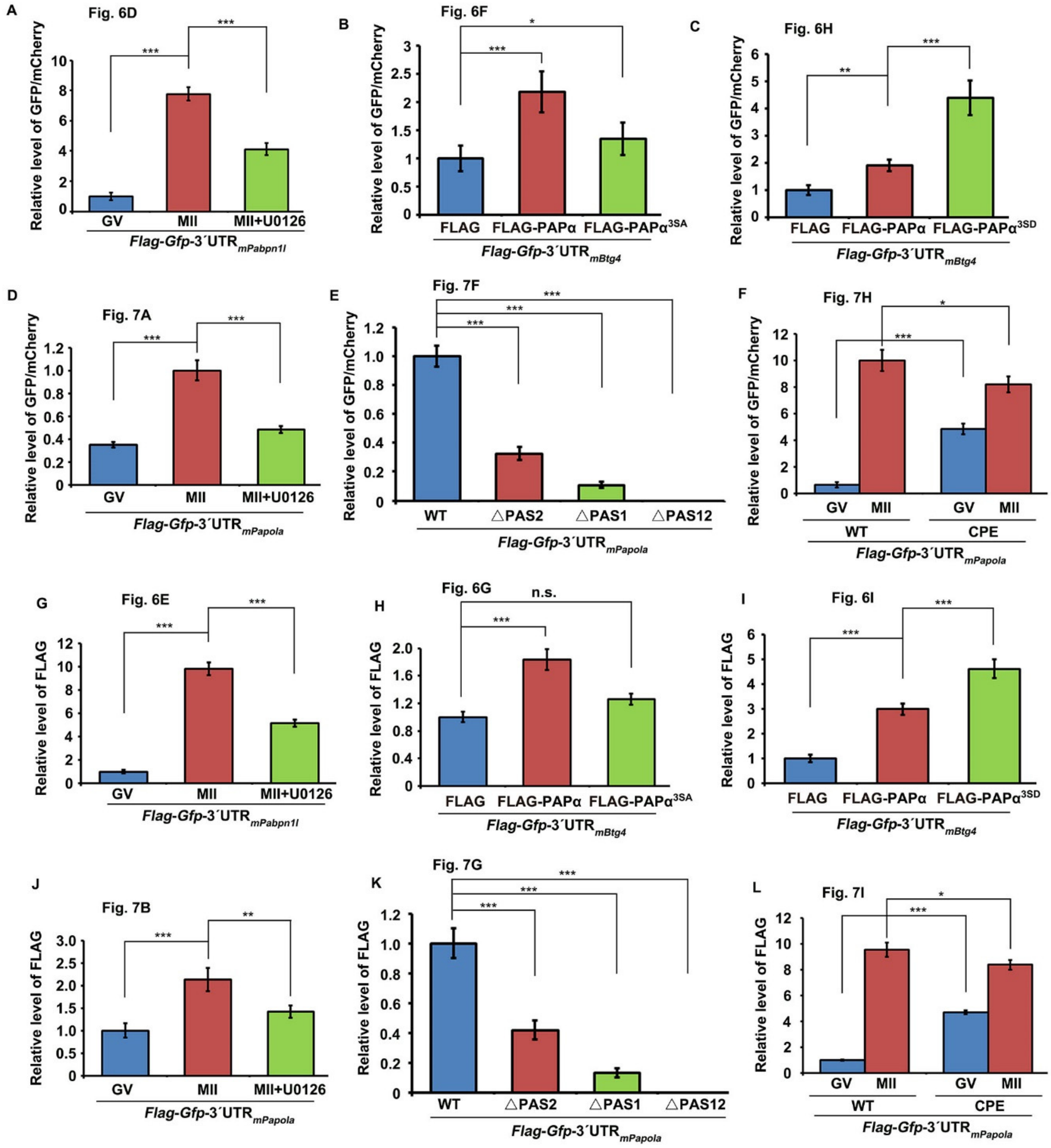

Supplementary Figure S3: Quantification of fluorescence signals in 3′-UTR reporter experiments. A–F: Fluorescence intensity of GFP relative to mCherry in indicated figure panels. **G-L**: The relative level of FLAG-GFP in indicated figure panels. Error bars, standard error of the mean (SEM). *$P < 0.05$, **$P < 0.01$, ***$P < 0.001$.

Supplementary Tables

Supplementary Table 1. Primer sequences.

**Primer name**	**Genes targeted**	**Application**	**Sequences (5′-3′)**
Papola-siRNA1	*Papola*	siRNA1-sense<br>siRNA1-antisense	5′-CCAUCAAACUGUGGGCCAATT-3′<br>5′-UUGGCCCACAGUUUGAUGGTT-3′
Papola-siRNA2		siRNA2-sense<br>siRNA2-antisense	5′-GCAUCAACUCUUGUACAUATT-3′<br>5′-UAUGUACAAGAGUUGAUGCTT-3′
R1	N.A.	Anchor primer for PAT assay	5′-GCGAGCTCCGCGGCCGCGTTTTTTTTTTTT-3′
Ccnb1-F	*Ccnb1*	PAT assay (with R1)	5′-CGCCACTCCTGTCTTGTAATGCCA-3′
Cnot7-F	*Cnot7*	PAT assay (with R1)	5′-TGGACTACAAGTTGTAATGTGTG-3′
Btg4-F	*Btg4*	PAT assay (with R1)	5′- GTAGGTTTTCAACTAAGGAAGAT-3′
Wee2-F	*Wee2*	PAT assay (with R1)	5′-GGCAGAGGACCCAGGAATTTTG-3′
Papola-F	*Papola*	PAT assay (with R1)	5′-CTAGTTTCCATAACCTGGAGATCAGACTGTT-3′
A0		PCR with *Papola* -F	5′-AGCTCTTGAAAAATACAAGGAGTACAGATACTATAAAAC-3′

Supplementary Table 2. Antibody information.

Protein name	Manufacturer (catalog number)	Applications (working dilution)	Website Link*
**PAPα**	Sigma-Aldrich (HPA001788)	WB(1:1000) IF (1:100)	https://www.sigmaaldrich.com/catalog/product/sigma/hpa001788?lang=zh&region=CN
**p-S558-PAPα**	This study	WB(1:1000)	N/A
**FITC-α-Tubulin**	Sigma-Aldrich (F2168)	WB(1:1000) IF (1:500)	http://www.sigmaaldrich.com/catalog/product/sigma/f2168?lang=zh&region=CN
**ERK1/2**	Santa Cruz (sc-94)	WB(1:1000)	http://www.scbt.com/datasheet-94-erk-1-k-23-antibody.html
**p-ERK1/2**	Cell Signaling (9101)	WB (1:1000)	http://www.cellsignal.com/products/primary-antibodies/phosphop44-42-mapk-erk1-2-thr202-tyr204-antibody/9101
**FLAG**	Sigma-Aldrich (F3165)	WB (1:2000)	http://www.sigmaaldrich.com/catalog/product/sigma/f3165?lang=zh&region=CN
**HA**	Cell Signaling (3724)	WB (1:2000)	http://www.cellsignal.com/products/primary-antibodies/ha-tagc29f4-rabbit-mab/3724
**DDB1**	Epitomics (3821-1)	WB (1:2500)	http://www.epitomics.com/products/search/DDB1
**CPEB1**	Proteintech (13274-1-AP)	WB (1:500)	https://www.ptglab.com/products/CPEB1-Antibody-13274-1-AP.htm
**Cyclin B1**	Cell Signaling (4138)	WB (1:1000)	https://www.cst-c.com.cn/products/primary-antibodies/cyclin-b1-antibody/4138
**CNOT7**	Santa Cruz (sc-101009)	WB (1:1000)	http://www.scbt.com/datasheet-101009-cnot7-18w-antibody.html
**BTG4**	Abcam (ab206914)	WB (1:1000)	http://www.abcam.com/btg4-antibody-eprzju-21-ab206914.html

WB, western blotting
